# A Quantum–Mechanical Study of Clean and Cr–Segregated Antiphase Boundaries in Fe_3_Al

**DOI:** 10.3390/ma12233954

**Published:** 2019-11-28

**Authors:** Martin Friák, Monika Všianská, Mojmír Šob

**Affiliations:** Institute of Physics of Materials, Czech Academy of Sciences, Žižkova 22, CZ-616 62 Brno, Czech Republic; vsianska@ipm.cz (M.V.); mojmir@ipm.cz (M.Š.)

**Keywords:** Fe3Al, antiphase boundaries, magnetism, ab initio, stability, chromium, segregation

## Abstract

We present a quantum-mechanical study of thermodynamic, structural, elastic, and magnetic properties of selected antiphase boundaries (APBs) in Fe${}_{3}$Al with the D0${}_{3}$ crystal structure with and without Cr atoms. The computed APBs are sharp (not thermal), and they have {001} crystallographic orientation. They are characterized by a mutual shift of grains by 1/2〈100〉*a* where *a* is the lattice parameter of a cube-shaped 16-atom elementary cell of Fe${}_{3}$Al, i.e., they affect the next nearest neighbors (APB-NNN type, also called APB-D0${}_{3}$). Regarding clean APBs in Fe${}_{3}$Al, the studied ones have only a very minor impact on the structural and magnetic properties, including local magnetic moments, and the APB energy is rather low, about 80 ± 25 mJ/m${}^{2}$. Interestingly, they have a rather strong impact on the anisotropic (tensorial) elastic properties with the APB-induced change from a cubic symmetry to a tetragonal one, which is sensitively reflected by the directional dependence of linear compressibility. The Cr atoms have a strong impact on magnetic properties and a complex influence on the energetics of APBs. In particular, the Cr atoms in Fe${}_{3}$Al exhibit clustering tendencies even in the presence of APBs and cause a transition from a ferromagnetic (Cr-free Fe${}_{3}$Al) into a ferrimagnetic state. The Fe atoms with Cr atoms in their first coordination shell have their local atomic magnetic moments reduced. This reduction is synergically enhanced (to the point when Fe atoms are turned non-magnetic) when the influence of clustering of Cr atoms is combined with APBs, which offer specific atomic environments not existing in the APB-free bulk Fe${}_{3}$Al. The impact of Cr atoms on APB energies in Fe${}_{3}$Al is found to be ambiguous, including reduction, having a negligible influence or increasing APB energies depending on the local atomic configuration of Cr atoms, as well as their concentration.

## 1. Introduction

Antiphase boundaries (APBs) are extended defects in crystals with multiple ordered sublattices. They separate two mutually shifted regions of the same ordered phase. The shift occurs during ordering processes, e.g., when different grains crystallize from different positions in the melt having a shifted origin defining their lattices. An APB is formed when two such domains meet and form an interface. As the formation of these interfaces occurs at elevated temperatures when diffusion and other thermal processes are active, an intermediate disordered phase can form (thermal APBs). Dislocations with Burgers vectors that are not translation vectors of the ordered superlattice can also create APBs, as they move through an ordered phase (deformation APBs with sharp interfaces).

**Figure 1:**
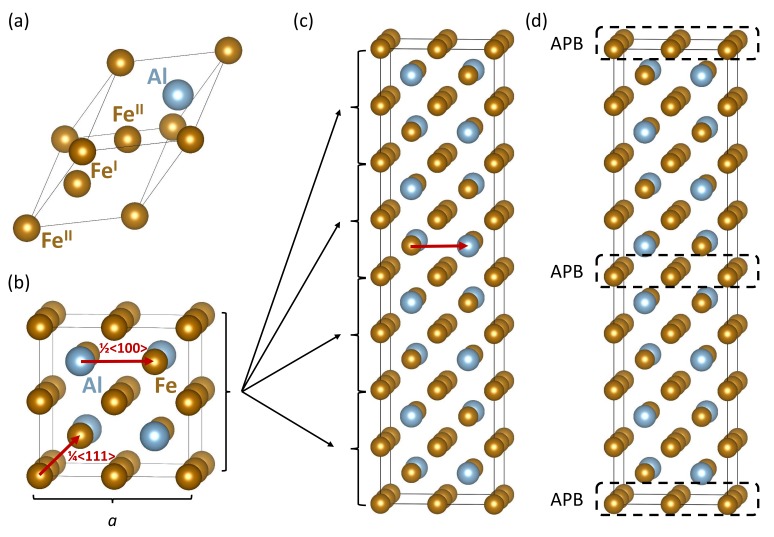
Schematic visualizations of computational cells related to the present study. A rhombohedral four atom primitive unit cell of Fe${}_{3}$Al is shown in (**a**) and includes the notation of Fe sublattices used in the present study (Fe${}^{\mathrm{II}}$ sites being twice more abundant than Fe${}^{I}$ sites). A 16-atom cube-shaped elementary cell with four formula units is visualized in (**b**) and includes two vectors defining two types of antiphase boundaries (APBs). A 64-atom supercell as a 1×1×4 multiple of the 16-atom elementary cell is shown in (**c**). When applying the 1/2〈100〉*a* shift APB vector (APB-next nearest neighbors (NNN)) to the upper half of the 64-atom supercell in (**c**), an APB-containing supercell shown in (**d**) is formed.

Our theoretical study is focused on APBs with sharp interfaces in Fe${}_{3}$Al (see its formula unit in Figure 1a) as a material belonging to a very promising class of Fe–Al materials planned for high-temperature applications. Members of this material family possess very interesting properties including remarkable resistance to oxidation, relatively low density, electrical resistivity, or rather low cost of raw materials [1,2,3]. A wider use of Fe–Al alloys is partly hindered by their lower ductility at ambient temperatures and a drop of the strength at elevated temperatures [3]. The ambient temperature brittleness was shown to be an extrinsic effect [4,5] and could be suppressed [6,7]. In recent decades, there has been intensive experimental research (see, e.g., [8,9,10,11,12,13,14,15,16,17]), as well as theoretical effort [18,19,20,21,22,23,24,25,26,27,28,29,30,31,32,33,34,35,36,37,38,39] focused on Fe–Al based materials and improvement of their properties.

A special sub-class of Fe–Al based materials is represented by superalloys, i.e., specific nano-composites consisting of two phases, ordered Fe${}_{3}$Al with the D0${}_{3}$ structure and a disordered Fe–Al solid solution with about 18–19 at. % Al. Our study was partly motivated by the fact that experimental evidence of the co-existence of Fe${}_{3}$Al and a disordered Fe–Al phase has been provided by analyzing APBs detected by the transmission electron microscopy (TEM) technique. In their classical papers, Marcinkowski and Brown observed APBs in thin foils of Fe-Al alloys by TEM [40,41]. They reported two types of APBs for the D0${}_{3}$ superlattice of Fe${}_{3}$Al (both types are involved in separating the partials of superdislocations in Fe-Al materials [42,43,44]). One of them appears in both the D0${}_{3}$ superlattice and in the B2 lattice (a higher-temperature phase) of Fe${}_{3}$Al and is characterized by a shift of the interfacing grains by 1/4〈111〉*a* where *a* is the lattice parameter of the cube-shaped 16-atom elementary cell of Fe${}_{3}$Al (shown in Figure 1b). It interrupts the chemical order of the first nearest neighbors (APB-NN type, also called APB-B2). The second APB type is specific to the D0${}_{3}$ superlattice, and it shifts the grains with respect to the each other by 1/2〈100〉*a* (see Figure 1b). It disturbs the chemical order of the next nearest neighbors (APB-NNN type, also called the APB-D0${}_{3}$ type). Other studies followed more recently [45,46,47,48,49]. As some parts of Fe${}_{3}$Al samples after cooling can exist in the higher temperature B2 lattice (and not the low temperature D0${}_{3}$ superlattice; see, e.g., [49]) and the APBs with 1/4〈111〉*a* can exist in both types of lattices, we focus our study on the D0${}_{3}$ type (APB-NNN) of APBs with the 1/2〈100〉*a* shift, which are specific to the D0${}_{3}$ lattice of Fe${}_{3}$Al.

Our study also includes Cr additions as some of the limitations associated with the binary Fe–Al system can be overcome by suitable alloying. In particular, McKamey [50] conducted a study of an alloy Fe–28 at. % Al and found that the room-temperature ductility can be improved by a factor of at least two by additions of chromium up to 6 at. %. Culbertson and Kortovich [51] reported results involving the addition of certain elements, including Cr, on the mechanical properties, workability, and oxidation resistance of Fe–Al. At 600 ${}^{o}$C, significant increases in yield strength were produced by additions of Si, Ta, Mo, Nb, or Cr. However, only an alloy containing additions of 3.3% Cr and 0.2% Mo (the matrix was Fe–23.4% Al) exhibited a room-temperature ductility close to 5% in Fe–Al.

## 2. Materials and Methods

Our calculations were performed with the help of the Vienna Ab initio Simulation Package (VASP) [52,53] implementing the density functional theory [54,55]. We used projector augmented wave (PAW) pseudopotentials [56,57]. The generalized gradient approximation (GGA) as parametrized by Perdew and Wang [58] (PW91) was employed for the exchange and correlation energy, and we also used the Vosko–Wilk–Nusair correction [59]. The plane-wave energy cut-off was equal to 400 eV, and the product of the number of Monkhorst–Pack k-points and the number of atoms was equal to 27,648 (e.g., 12 × 12 × 3 k-point mesh in the case of the 64-atom 1 × 1 × 4 multiple of the cube-shaped 16-atom elementary cell of Fe${}_{3}$Al). When computing a complete set of elastic constants $C_{ij}$, we applied the stress-strain method [60]. All but one calculated state were initially set up as ferromagnetic with all local magnetic moments with parallel orientation (the only exception is discussed in Appendix A).

## 3. Results

When simulating the D0${}_{3}$ specific type of APBs (APB-NNN) with the {001} interface plane, we computed the properties of the 64-atom supercell of defect-free Fe${}_{3}$Al (Figure 1c) and that containing the APB (Figure 1d). As the APB energy typically depends on the crystallographic orientation of the interface only very weakly, we suppose that our choice of the {001} interface plane was sufficiently representative. The energy of the APB-containing supercell with the two identical APBs was higher than that of defect-free Fe${}_{3}$Al by 327 meV, i.e., only 5.1 meV per atom. As the energy difference was so small, we also repeated our calculations with the cut-off energy equal to 600 eV, as well as with eight times more k-points (24 × 24 × 6) with the cut-off energy equal to 400 eV and 600 eV and the energy difference changed only by less than 1% of the above-mentioned value of 5.1 meV/atom. The lattice parameters within the {001} plane were equal to 5.713 Å, i.e., 0.32% smaller than in the case of the defect-free Fe${}_{3}$Al, 5.731 Å. The APB energy was then equal to 80 mJ/m${}^{2}$. In order to test the interactions between APBs, we performed calculations using supercells with the same size within the {001} plane, but different sizes in the [001] direction. They contained 32, 48, 96, and 192 atoms (having a smaller or bigger distance between the APBs), and the APB energies were equal to 60 mJ/m${}^{2}$, 71 mJ/m${}^{2}$, 63 mJ/m${}^{2}$, and 105 mJ/m${}^{2}$, respectively. We estimated the APB energy to be 80 ± 25 mJ/m${}^{2}$. The interactions between the APBs could be long-range, but we suppose that our study based on systematically using 64-atom (or 256-atom) supercells captured the main trends correctly.

Regarding magnetic properties, the APBs have only very little impact on the local magnetic moments of atoms (the changes are in the second digit behind the comma), and the total magnetic moment of the defect-free Fe${}_{3}$Al, 95.1 $\mu_{B}$ per 64 atoms, was only slightly higher than that of the APB-containing supercell, 94.6 $\mu_{B}$. This small and negative change of the magnetization was in contrast with our recent findings related to APBs in the off stoichiometric variant of Fe${}_{2}$TiAl phase [61] where a nearest-neighbor type of APB (APB-NN) resulted in a huge increase of the total magnetic moments by 140%. This increase was due to (i) the fact that APBs become increasing disordered in Fe${}_{2}$TiAl and, in this way, allow Fe atoms to be surrounded by less Al atoms (which lower the magnetic moments of Fe atoms [20]) and (ii) a change from a ferrimagnetic state to a ferromagnetic one. Two reasons for a much lower impact of APBs in the present study could be (i) our choice of a different type of APB and, certainly, (ii) a different material, Fe${}_{3}$Al without any Ti atoms (as in [61]).

Interestingly, while the APB-induced changes in the energy and magnetic moments were relatively small, elastic properties, as a representative of tensorial materials’ characteristics, were much more clearly affected. The 6 × 6 matrices of the elastic constants of defect-free Fe${}_{3}$Al (Figure 1c) and the APB-containing one (Figure 1d) as obtained from the stress-strain method are:

$$\begin{array}{c} {Fe}_{3}Al:\left(\begin{array}{cccccc} 212 & 162 & 162 & 0 & 1 & 0\\ 162 & 212 & 160 & 1 & 0 & 0\\ 162 & 160 & 212 & 0 & -1 & -2\\ 0 & 1 & 0 & 139 & 0 & 0\\ 1 & 0 & -1 & 0 & 139 & 0\\ 0 & 0 & -2 & 0 & 0 & 140 \end{array}\right),\;{Fe}_{3}Al-APB:\left(\begin{array}{cccccc} 211 & 163 & 155 & 0 & 0 & 0\\ 163 & 211 & 153 & 1 & 0 & 0\\ 155 & 153 & 187 & 0 & -1 & -2\\ 0 & 1 & 0 & 138 & 0 & 0\\ 0 & 0 & -1 & 0 & 137 & 0\\ 0 & 0 & -2 & 0 & 0 & 139 \end{array}\right). \end{array}$$

**Figure 2:**
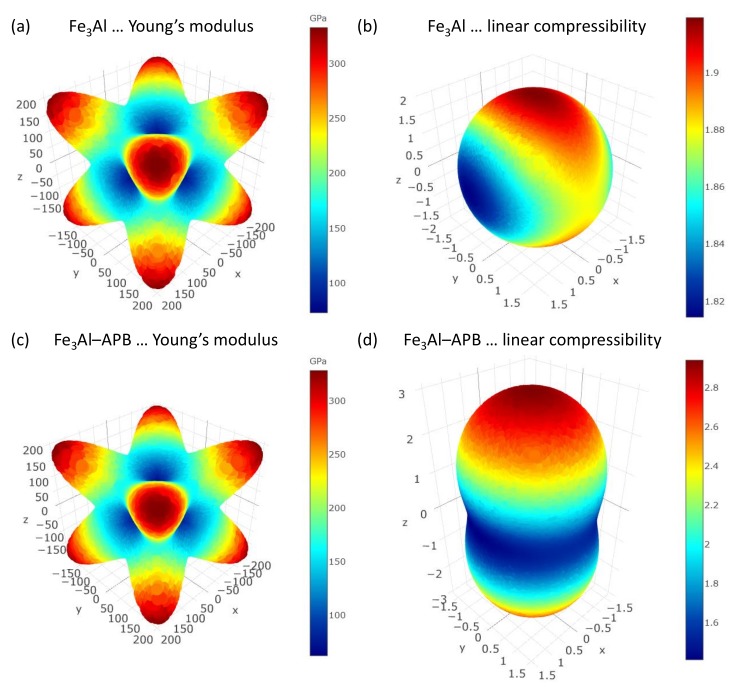
Computed elastic properties of Fe${}_{3}$Al with and without the studied type of APBs (visualized in Figure 1d). The elasticity is shown in the form of the directional dependences of the Young’s modulus (**a**,**c**) and linear compressibility (**b**,**d**) for the APB-free Fe${}_{3}$Al (**a**,**b**), as well as for the Fe${}_{3}$Al with APBs (**c**,**d**), respectively. Mind different scales in the case of linear compressibilities (**b**,**d**). The visualization was performed with the help of the MELASA software [62], https://melasa.cerit-sc.cz/.

The elastic properties of both the defect-free and APB-containing Fe${}_{3}$Al are also visualized in the form of the directional dependences of Young’s modulus and linear compressibility in Figure 2. It is worth mentioning that the defect-free Fe${}_{3}$Al (Figure 1c) has a cubic symmetry, and therefore, if the coordinate system was chosen along the unit cell vectors, it should be characterized by $C_{11}$ = $C_{22}$ = $C_{33}$, $C_{12}$ = $C_{13}$ = $C_{23}$, $C_{44}$ = $C_{55}$ = $C_{66}$ and all other elastic constants equal to zero. The fact that these relations were not quite fulfilled in the left 6 × 6 matrix above was caused by numerical errors when computing the elastic constants by the stress–strain methods. We list them in order to demonstrate the error bar of our calculations (1–2 GPa). If all the cubic-symmetry relations were valid, the linear compressibility of the defect-free Fe${}_{3}$Al (see Figure 2b) would be a sphere. Due to numerical errors, we saw a small deviation from a spherical shape. As far as the impact of APBs was concerned, the simulated APBs changed the symmetry of the elastic properties to a tetragonal one (as seen in Figure 2d). The linear compressibility was much more sensitive to this qualitative difference (Figure 2d vs. Figure 2b), while the directional dependences of the Young’s modulus in Figure 2a,c do not show it so clearly.

The loss of the cubic symmetry was quantified by the difference between the values of elastic constants $C_{11}$ = $C_{22}$ = 211 GPa and $C_{33}$ = 187 GPa in the case of the APB-containing system. It should be noted that the effect will very likely be decreasing for a decreasing density of APBs (similar to the case of the elastic properties of grain boundaries in general; see, e.g., [63,64,65]). The APB density simulated in this study was equal to two per 64-atom supercell containing sixteen {001} planes. It is worth noting that the impact of APBs on the elastic properties would be very difficult to capture by a typical linear elasticity method, such as that of Grimsditch and Nizzoli [62,66], because they treated both shifted grains as the same elastic continuum (it was very difficult to include the APB shift).

**Figure 3:**
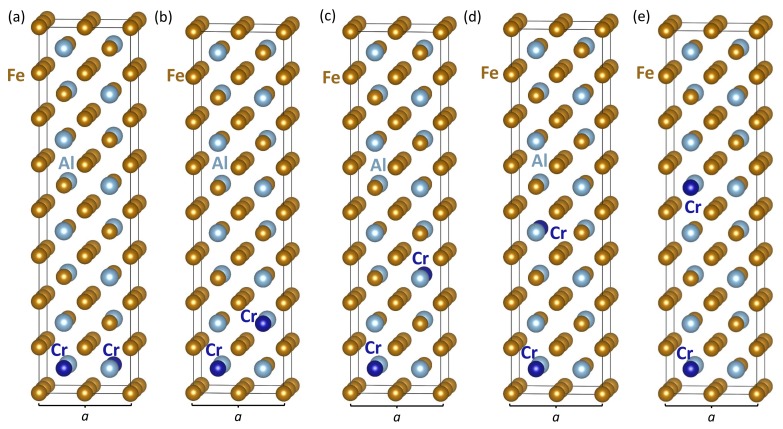
Atomic configurations of two Cr atoms per 64-atom supercell of defect-free Fe${}_{3}$Al. The Cr atoms are at Fe${}^{I}$ positions within a single {001} plane (**a**), separated by one {001} plane of Fe${}^{\mathrm{II}}$ atoms (**b**), three {001} planes (**c**), and by five {001} planes (**d**). The rightmost 64-atom supercell (**e**) is that with two Cr atoms as far from one another as possible within our 64-atom supercell (but, mind the periodic images in the distance of the lattice parameter *a* within the {001} plane).

Before analyzing the impact of Cr atoms on the properties of APBs, we focus on interactions of Cr atoms in APB-free Fe${}_{3}$Al. In order to do so, we compared the thermodynamic and magnetic properties of five different distributions of Cr atoms in our 64-atom supercells visualized in Figure 3. The Cr atoms were located at the Fe${}^{I}$ sublattice because these positions were energetically preferred over the Fe${}^{\mathrm{II}}$ ones by 467 meV per 64-atom supercell (see the detailed comparison in Appendix A), as well as over the Al sites (see, e.g., results tabulated in Ref. [67]). Our set of structures included the smallest possible distance (see Figure 3a), as well as more separate ones (Figure 3b–e)) (mind the periodic images within the {001} plane in the distance of one lattice constant *a*, which is defined in Figure 3). As we had two APBs per 64-atom supercell, we limited our study to two Cr atoms in 64-atom supercells.

The calculated energies and magnetic moments of different configurations of Cr atoms in the Fe${}_{3}$Al are summarized in Table 1. Our results clearly show that Cr atoms tend to cluster. The energy gain was about 105 meV per two Cr atoms when comparing the configuration with the Cr atoms as close as possible (Figure 3a) and that when the Cr atoms were far apart (Figure 3e). Again, it is worth noting the impact of the periodic boundary conditions. First, a whole Cr–Al plane without any Fe atoms was formed in the case shown in Figure 3a. Second, all other configurations (Figure 3b–e) contained {001} planes with 25 at. %Cr (with periodic images of Cr atoms within the {001} plane in the distance of one lattice constant *a*) with a different distance between the {001} plane.

Importantly, the Cr atoms tended to transform the ferromagnetic state of Fe${}_{3}$Al into a ferrimagnetic one as the local magnetic moments of Cr atoms were antiparallel to those of the Fe atoms. Consequently, the total magnetic moments $\mu^{\mathrm{TOT}}$ of the 64-atom Cr-containing supercells, which were equal to values from 81.0 to 82.6 $\mu_{B}$ (see Table 1), were significantly lower than that of the 64-atom supercell of Fe${}_{3}$Al, which was equal to 95.1 $\mu_{B}$. The reduction of the total magnetic moment in the case of the Cr-containing Fe${}_{3}$Al structures was, in fact, an interesting interplay of a few different mechanisms described below.

The first reduction mechanism was related to the fact that the Cr atoms in the supercells shown in Figure 3 substituted iron atoms at the Fe${}^{I}$ sublattice, which were, in the defect-free Fe${}_{3}$Al, in high spin states with local magnetic moments of 2.4 $\mu_{B}$. These contributions to the total magnetic moment were missing in the Cr-containing systems. Second, the local magnetic moments of Cr atoms were anti-parallel, and the total magnetization was thus reduced by their magnitudes (see the negative values in Table 1). Moreover, this magnitude changed, depending on the atomic configuration, from 0.9 to 1.3 $\mu_{B}$. The third aspect was related to Fe atoms surrounding the Cr atoms and was found to be very sensitive to the local atomic configuration, as well. Table 1 summarizes the local magnetic moments of Fe atoms at both iron sublattices. The iron atoms on the Fe${}^{I}$ sublattice, which never had any Cr atom in the first nearest neighbor shell, had quite similar values of the local magnetic moment between 2.3 and 2.5 $\mu_{B}$. These values were close to 2.4 $\mu_{B}$ computed for the Cr-free Fe${}_{3}$Al. In contrast, iron atoms on the Fe${}^{\mathrm{II}}$ sublattice had their local magnetic moments significantly reduced (from 1.9 $\mu_{B}$ computed for the Cr-free Fe${}_{3}$Al) when having Cr atoms in their first nearest neighbor shell (1NN). When the Fe${}^{\mathrm{II}}$ atoms had two Cr atoms in their 1NN (see Figure 3a,b), their local magnetic moments were reduced to 0.8–0.9 $\mu_{B}$. When there was only one Cr atom in the 1NN, the local magnetic moments of Fe${}^{\mathrm{II}}$ atoms were lowered to 1.5–1.6 $\mu_{B}$.

**Figure 4:**
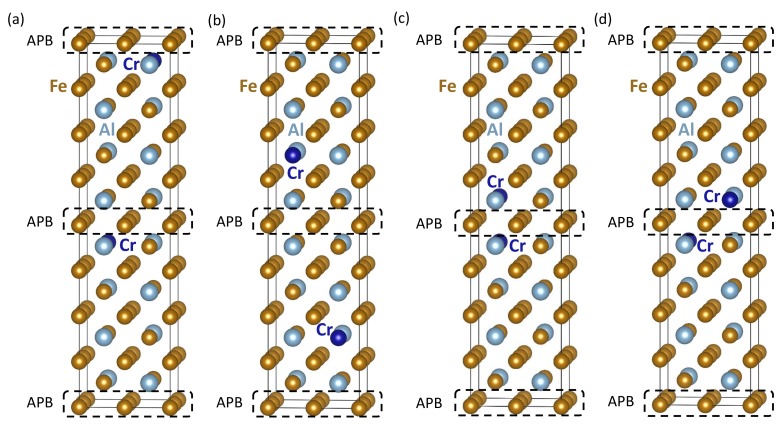
Visualizations of 64-atom supercells of APB-containing Fe${}_{3}$Al structures with two Cr atoms per supercell. Part (**a**) shows the case of two identical APBs with one Cr atom close to each of them, while (**b**) is an example of the distribution of Cr atoms that are as far as possible (within our supercells) from the two APBs, i.e., three and five {001} atomic planes from the APBs. Parts (**c**,**d**) represent two cases with both Cr atoms segregated to one of the APBs, while the other APB has no Cr atom nearby.

Next, we examine the impact of Cr atoms on APBs in Fe${}_{3}$Al. The studied configurations are shown in Figure 4. We compare a series of atomic configurations with two Cr atoms occupying positions on the Fe${}^{I}$ sublattice in different atomic configurations of the two Cr atoms.

When evaluating the APB energy in Fe${}_{3}$Al structures containing Cr atoms, there is an important question related to the proper reference state. The atomic configuration shown in Figure 4a has one Cr atom as close as possible to each of the two APBs, and these APBs (and Cr atoms) are as far away from one another as possible in our 64-atom supercell. If we chose the configuration in Figure 3e as the reference state (each of the two Cr atoms as far as possible from one another), then the APB energy, 79.5 mJ/m${}^{2}$, was only very slightly lower than in the Cr-free case discussed above (with the APB energy equal to 80 mJ/m${}^{2}$). As the difference between the APB energies of the Cr-free and Cr-containing case was, in fact, within an expected error bar of our calculations, we would rather conclude that the APB energy was not affected by Cr atoms in this particular atomic configuration.

As far as the structure is concerned, the total volume of the 64-atom supercell (Figure 4a) was 748.09 Å${}^{3}$, and the lattice parameter within the APB {001} was equal to 5.717 Å, i.e., nearly identical to the lattice parameter 5.713 Å (and slightly smaller than the total volume of 753.20 Å${}^{3}$) in the case of the APB-free Fe${}_{3}$Al discussed above. However, the two compared configurations of Cr atoms, the APB-free one in Figure 3e and that with APBs in Figure 4a, were those with the maximum distance between the Cr atoms. Again, it is worth emphasizing that the nearest Cr neighbors of the Cr atoms were in the configurations of Figure 3e and Figure 4a periodic images within the {001} plane at a distance of one lattice constant *a*.

Let us now analyze the segregation tendencies of Cr atoms with respect to the studied APBs. As we will see below, the situation was rather complex. A first indication can be deduced from the comparison of the energies of the atomic configurations visualized in Figure 4a,b. The latter (Figure 4b) corresponds to the situation when each of the two Cr atoms was as far as possible from one another and also as far as possible from each of the two APBs. The configuration in Figure 4b with the Cr atoms away from APBs has an energy 20 meV per 64-atom supercell lower than the configuration in Figure 4a. This fact can be interpreted as a thermodynamic driving force (of 10 meV per Cr atom) to move the Cr atoms away from APBs, i.e., opposite of the segregation tendency of Cr atoms towards APBs.

On the other hand, if we analyze the configurations visualized in Figure 4c,d, the trends turned out to be qualitatively opposite. These two configurations represent two cases when both Cr atoms in our supercells were located very close one to another and next to one of the two APBs. The atomic configuration in Figure 4c with the two Cr atoms as close as possible had energy lower than that in Figure 4a by 189 meV per 64-atom supercell. When keeping the Cr atoms still as close as possible to the APB, but locating them further apart from one another (see Figure 4d), the energy gain (with respect to the configuration in Figure 4a) was much lower, only 30 meV per 64-atom supercell. This comparison would indicate that the tendency of Cr atoms to cluster was much stronger than the tendency to segregate to APBs. However, this conclusion is closely related to one specific configuration in Figure 4c.

The pairs of Cr atoms located as close as possible across the APBs would form thermodynamically very stable configurations. Such thermodynamically stable APB-related atomic configurations of Cr atoms can be candidate structures for specific APB interface states, which are under similar circumstances, close to (extended) defects, sometimes called complexions [68,69,70,71,72,73,74,75,76,77,78,79,80,81]. The impact of APBs is critically important because the two configurations of Cr atoms close to the APB shown in Figure 4c,d are both APB-specific and would not occur in a APB-free bulk Fe${}_{3}$Al. Therefore, below, we closely analyze their magnetic properties, in particular the local magnetic moments of Fe atoms. Our results are summarized in Table 2.

It is possible to distinguish the cases in Figure 4a,b when each Cr atom is so far away from the others that there is no Fe atom that would have them both in its first nearest neighbor shell (1NN). The Fe${}^{\mathrm{II}}$ atoms with one Cr atom in their first nearest neighbor coordination shell had their magnetic moments reduced from the Fe${}_{3}$Al bulk value of 1.9 $\mu_{B}$ to 1.4–1.6 $\mu_{B}$. In contrast to the two structures in Figure 4a,b, when Cr atoms were close to each other (see Figure 4c,d), both of them could be in the first coordination sphere of some Fe${}^{\mathrm{II}}$ atoms. These then had their local magnetic moments reduced to as low as 0.5 $\mu_{B}$ (see the values in Table 2 for Figure 4c) or even completely suppressed (see the last row of data in Table 2 for the configuration in Figure 4d). The Cr atoms themselves had the magnitude of their local atomic moments between 0.9 and 1.3 $\mu_{B}$ and the orientation antiparallel to those of Fe atoms. The states were, therefore, again ferrimagnetic (rather than ferromagnetic in the case of bulk Fe${}_{3}$Al with or without the studied APBs). The presence of APBs seemed to have only a limited impact of the ferrimagnetic nature of the studied states. On the other hand, the effect of APBs could synergically combine with that of Cr atoms, giving rise to Fe atoms with an extremely low, even zero, magnitude of local magnetic moments close to APB specific atomic configurations not existing in an APB-free bulk.

As far as the APB energy is concerned in the case of the states visualized in Figure 4c,d, it is not possible to evaluate this directly as the atomic configurations of the two APBs in the supercells in Figure 4c,d are not equal. One of APBs had two Cr atoms, one on each side, while the other APB had no Cr atom nearby. Despite these differences, we could evaluate the average of APB energies of the two different APBs in each of the two supercells in Figure 4c,d. The choice of an APB-free reference state was in these two cases even more important than before. In order to address the identified clustering tendencies of Cr atoms, we evaluated the averaged APB energies with respect to three different states of Cr atoms in APB-free Fe${}_{3}$Al; in particular, the case with the two Cr atoms as far as possible (see Figure 3e) and then two states with Cr atoms very close one to another (in Figure 3a,b). The averaged interface energies of the configuration shown in Figure 4c were then equal to 34 mJ/m${}^{2}$, 56 mJ/m${}^{2}$, and 46 mJ/m${}^{2}$ with the references states in Figure 3e and Figure 3a,b, respectively. The most Cr clustered and APB-segregated structure shown in Figure 4c had average APB energies significantly lower than that associated with the configuration in Figure 4a when the Cr atoms were close to APBs, but far away from each another. More importantly, these lower averaged APB energies were also lower than those discussed above in the case of APBs in Cr-free Fe${}_{3}$Al. We would then conclude that Cr atoms lowered the APB energy. Contrary to that, rather moderate relocation of one of the Cr atoms further apart into the atomic configuration in Figure 4d resulted in an increase of the average APB energies (even higher than those obtained above for Cr-free Fe${}_{3}$Al). The average APB energies in the case of the configuration in Figure 4d were 73 mJ/m${}^{2}$, 95 mJ/m${}^{2}$, and 85 mJ/m${}^{2}$ with the references states in Figure 3e and Figure 3a,b, respectively. The actual impact of Cr atoms on the energetics of APBs in Fe${}_{3}$Al was thus very sensitive to the configuration of Cr atoms (they could both increase or decrease the APB energies). This qualitatively agrees with conflicting experimental reports of a reduction [42], increase [44], or no impact [43] of Cr atoms on APB energies in a slightly different material, Fe-28 at. % Al [67].

**Figure 5:**
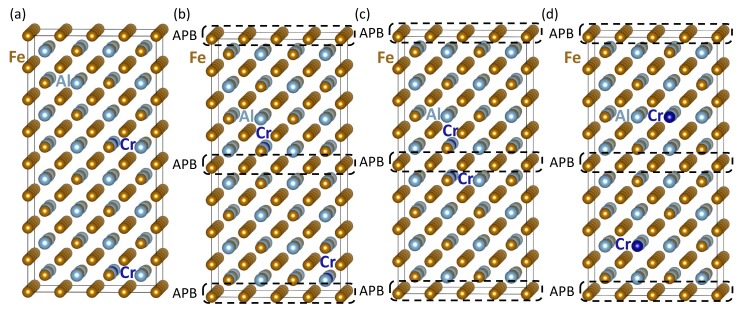
Schematic visualization of 256-atom supercells used in our calculations with lower concentrations of Cr atoms (2/256, i.e., 0.78125 at. %). An APB-free Fe${}_{3}$Al structure with two Cr atoms very far apart (**a**). The case of two identical APB configurations, each APB with one Cr atoms close to it (**b**). An APB state with two Cr atoms as clustered as possible and as close to one of the two APBs as possible (**c**). An atomic configuration with both Cr atoms mutually far apart and also far from APBs (**d**).

Finding the APB energies so sensitive to the local distribution of Cr atoms, it also became clear that there was another aspect of the problem that needed to be considered, namely our choice of the shape and size of supercells. The shape of 64-atom supercells combined with the application of periodic boundary conditions led to a rather high planar concentration of Cr atoms within the {001} planes, typically 25% (but also twice more in the case shown in Figure 3a), nearest neighbors of Cr atoms being represented by their own periodic images within the {001} plane (configurations in Figure 3c–e and Figure 4a,b). The size of our supercells also determined the concentration of Cr atoms to be 2/64, i.e., 3.125 at. %. In order to test how sensitive our results were to these aspects, we also simulated APBs with and without Cr atoms in 256-atom supercells with the geometry a 2 × 2 × 4 multiple of cube-shaped 16-atom elementary cell of Fe${}_{3}$Al (see Figure 1b). The 256-atom supercells used are shown in Figure 5. The comparison of an APB-free Fe${}_{3}$Al structure in Figure 5a and that with two equal APBs each with one Cr atom as close to the APB plane as possible (see Figure 5b) allowed for the determination of the APB energy. The computed value 72 mJ/m${}^{2}$ was about 10% lower than that in the case of the higher Cr concentration shown in Figure 4a, 80 mJ/m${}^{2}$, and in the Cr-free Fe${}_{3}$Al case (Figure 1d), 80 mJ/m${}^{2}$. The Cr atoms in this configuration thus reduced the APB energy.

When checking the atomic configuration in Figure 5c,d, it is interesting to find that the energy increased by 65 meV and 74 meV per 256-atom supercell in Figure 5c,d, respectively, with respect to the configuration in Figure 5b. The location of two Cr atoms to be as close as possible (Figure 4c) was thus not always energetically preferred. This complexity very likely illustrates the role of lateral interactions of Cr atoms within the {001} plane. When there was only a single Cr atom per one {001} plane in Figure 5, the planar concentration of Cr atoms in Figure 5 was only 1/16, i.e., 6.25 at. %, and the lateral interactions with other Cr atoms were significantly reduced. Then, the atomic configuration with a single Cr atom close to the {001}-plane APB (Figure 5b) was preferred over the case with Cr atoms being far away from the APB planes in Figure 5d, which is in line with the reduction of APB energy due to the Cr atoms in the case of 256-atom supercells. The configuration in Figure 5b was also preferred over the configurations when there were two Cr atoms close to the APB plane, each on one side, and located mutually as close as possible in an APB specific configuration; see Figure 5c. We can thus conclude that the impact of Cr atoms on the APB energy in Fe${}_{3}$Al was sensitive not only to the actual atomic distribution, but also to the concentration of Cr atoms.

## 4. Conclusions

Our first-principles study was focused on the energetic, structural, and magnetic properties of clean and Cr-segregated antiphase boundaries (APBs) in Fe${}_{3}$Al with the D0${}_{3}$ crystal structure. We computed the characteristics of atomically sharp APBs with the {001} crystallographic orientation employing 64-atom and 256-atom supercells with or without two atoms of Cr per supercell. The studied APBs were characterized by a mutual shift of grains by 1/2〈100〉*a* where *a* is the lattice parameter of cube-shaped 16-atom elementary cell of Fe${}_{3}$Al, i.e., they affected the next nearest neighbors (APB-NNN). When computing the properties of APBs in Fe${}_{3}$Al, we found that they had only a very small impact on the structural and magnetic properties. The local magnetic moments of the Fe atoms were only marginally changed by APBs, and the APB energy was rather low, about 80 ± 25 mJ/m${}^{2}$. A stronger impact of APBs was predicted in the case of anisotropic (tensorial) elastic properties. The APBs induced a change from cubic symmetry to tetragonal symmetry, and this change was very sensitively reflected by the directional dependence of linear compressibility.

Regarding the Cr-containing structures, the Cr atoms had a very significant impact on the magnetic properties and energetics of APBs, but this influence was rather complex. As far as the magnetism is concerned, the Cr atoms exhibited clustering tendencies in Fe${}_{3}$Al, both without and with APBs, and caused a transition from a ferromagnetic state (of Cr-free Fe${}_{3}$Al) into a ferrimagnetic one. The Fe atoms with Cr impurities in their first coordination shell had their local atomic magnetic moments reduced. This reduction was synergically enhanced (to the point where some Fe atoms were turned completely non-magnetic) when the influence of clustering Cr atoms was combined with APBs, which offered specific atomic environments not existing in the APB-free bulk Fe${}_{3}$Al. As far as the impact of Cr atoms on APB energies in Fe${}_{3}$Al is concerned, it included a negligible effect, as well as both a reduction and increase of APB energies, depending on the local atomic configuration of Cr atoms, as well as their concentration. We hope that our results shed new light on a long lasting experimental controversy related to the impact of Cr atoms on APB energies in Fe-Al materials.

## Figures and Tables

**Table 1:** Calculated energies and magnetic moments of 64-atom supercells of Fe${}_{3}$Al without any APB containing two Cr atoms at different distances. The energies are listed relatively with respect to the atomic configuration with the minimum energy (Figure 3a). The local magnetic moments are provided for Cr atoms (negative values indicate their antiparallel orientation with respect to the orientation of Fe atoms) and both types of Fe sites. The local magnetic moments of Fe${}^{\mathrm{II}}$ atoms are also linked to the number of Cr atoms in their first coordination shell (1NN).

Computed Properties	Figure 3a	Figure 3b	Figure 3c	Figure 3d	Figure 3e
ΔE (meV per 64 atoms)	0	40	73	82	105
$\mu^{\mathrm{TOT}}$ ($\mu_{B}$ per 64 atoms)	81.0	81.8	82.6	82.6	82.4
$\mu^{\mathrm{Cr}}$ ($\mu_{B}$)	−0.9	−1.1	−1.3	−1.3	−1.3
$\mu^{\mathrm{Fe}}$ ($\mu_{B}$) Fe${}^{I}$	14 × (2.3–2.5)	14 × (2.3–2.5)	14 × (2.3–2.5)	14 × (2.3-2.5)	14 × (2.3–2.5)
$\mu^{\mathrm{Fe}}$ ($\mu_{B}$) Fe${}^{\mathrm{II}}$ no Cr in 1NN	24 × (1.8–1.9)	20 × (1.8–1.9)	16 × (1.8–1.9)	16 × (1.8-1.9)	16 × (1.8–1.9)
$\mu^{\mathrm{Fe}}$ Fe${}^{\mathrm{II}}$ with 1 Cr in 1NN		8 × 1.5	16 × (1.5–1.6)	16 × 1.5	16 × 1.5
$\mu^{\mathrm{Fe}}$ Fe${}^{\mathrm{II}}$ with 2 Cr in 1NN	8 × 0.9	4 × 0.8			

**Table 2:** Calculated energies and magnetic moments of 64-atom supercells of Fe${}_{3}$Al with APBs containing two Cr atoms in different atomic configurations. The energies are listed relatively with respect to the atomic configuration in Figure 4a. The local magnetic moments are provided for Cr atoms (negative values indicate their antiparallel orientation with respect to the orientation of Fe atoms) and both types of Fe sites. The local magnetic moments of Fe${}^{\mathrm{II}}$ atoms are also linked to the number of Cr atoms in their first coordination shell (1NN).

Computed Properties	Figure 4a	Figure 4b	Figure 4c	Figure 4d
ΔE (meV per 64 atoms)	0	−20	−189	−30
$\mu^{\mathrm{TOT}}$ ($\mu_{B}$ per 64 atoms)	82.4	82.5	80.8	77.2
$\mu^{\mathrm{Cr}}$ ($\mu_{B}$)	−1.2	−1.2	−0.9	−1.3
$\mu^{\mathrm{Fe}}$ ($\mu_{B}$) Fe${}^{I}$	14 × (2.3–2.6)	14 × (2.3–2.5)	14 × (2.3–2.6)	14 × (2.3–2.4)
$\mu^{\mathrm{Fe}}$ ($\mu_{B}$) Fe${}^{\mathrm{II}}$ no Cr in 1NN	16 × (1.8–1.9)	16 × (1.8–1.9)	20 × (1.8–1.9)	20 × (1.8–1.9)
$\mu^{\mathrm{Fe}}$ Fe${}^{\mathrm{II}}$ with 1 Cr in 1NN	16 × (1.4–1.6)	16 × 1.5	8 × (1.5–1.6)	8 × 1.4
$\mu^{\mathrm{Fe}}$ Fe${}^{\mathrm{II}}$ with 2 Cr in 1NN			4 × 0.5	4 × 0.0

**Figure A1:**
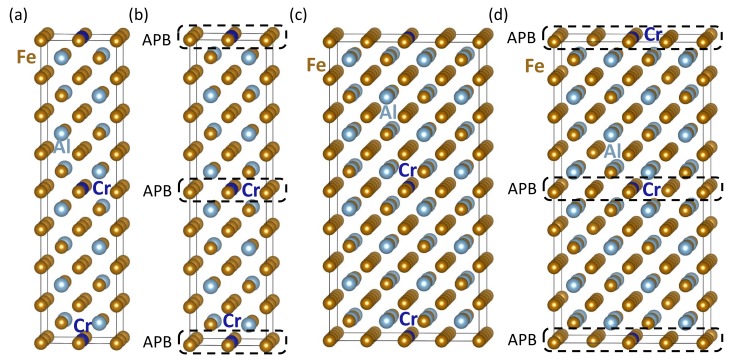
The computed atomic configurations of Cr atoms on the Fe${}^{\mathrm{II}}$ sublattice in the case of 64-atom supercells (**a,b**) and 256-atom ones (**c,d**) in the case of APB-free Fe${}_{3}$Al bulk (**a,c**), as well as APB-containing configurations (**b,d**).

## Appendix A

Our consideration of the Cr atoms solely in different Fe${}^{I}$ positions in the main text above was motivated by the higher energies that we obtained for Cr atoms located in the Fe${}^{\mathrm{II}}$ sublattice. In particular, we considered the atomic configurations shown in Figure A1. As far as 64-atom supercells of Cr-containing Fe${}_{3}$Al are concerned, the energy corresponding to two Cr atoms at the Fe${}^{\mathrm{II}}$ positions (Figure A1a) was higher by 505 meV per 64-atom supercell than the energy of the configuration in Figure 3e when both Cr atoms were in the Fe${}^{I}$ positions. When introducing {001}-plane APBs into the 64-atom supercell, the energy of the configuration when one Cr atom was directly within each of APB planes in Fe${}^{\mathrm{II}}$ positions (see Figure A1b) was higher by 590 meV than when there was one Cr atom close to each of the two APB planes, as visualized in Figure 4a. When reducing the Cr concentration by using 256-atom supercells, the situation was similar. The energy of the two Cr atoms located in Fe${}^{\mathrm{II}}$ positions in APB-free Fe${}_{3}$Al bulk, visualized in Figure A1c, was higher by 229 meV per 256-atom supercell than the configuration when the Cr atoms were in Fe${}^{I}$ positions; see Figure 5a. With one Cr atom in a Fe${}^{\mathrm{II}}$ position directly within each APB plane (see Figure A1d), the energy was higher by 705 meV than in the configuration in Figure 5b. The configuration in Figure A1d was started in a ferrimagnetic state with Cr atoms having their local magnetic moments antiparallel to those of the Fe atoms. The reason for this set up was that the initial ferromagnetic state converged into a state with a ferromagnetic orientation of local magnetic moments of Cr atoms, but this final state had yet higher energy (an additional 265 meV per 256-atom supercell).
